# Feasibility and safety of exercise training and nutritional support prior to haematopoietic stem cell transplantation in patients with haematologic malignancies

**DOI:** 10.1186/s12885-020-07637-z

**Published:** 2020-11-24

**Authors:** Erik Rupnik, Matevz Skerget, Matjaz Sever, Irena Preloznik Zupan, Maja Ogrinec, Barbara Ursic, Natasa Kos, Peter Cernelc, Samo Zver

**Affiliations:** 1 Department of Hematology, University Medical Centre Ljubljana, Zaloska cesta 7, 1000 Ljubljana, Slovenia; 2 Medical Faculty, University of Ljubljana, Vrazov trg 2, 1000 Ljubljana, Slovenia; 3 Institute for Medical Rehabilitation, University Medical Centre Ljubljana, Zaloska cesta 7, 1000 Ljubljana, Slovenia

**Keywords:** Haematopoietic stem cell transplantation, Prehabilitation, Physical performance, Body composition, Quality of life

## Abstract

**Background:**

Prehabilitation with regular exercise and nutritional care for patients undergoing surgeries for malignant disease was recently introduced to increase physiologic reserve prior to the procedure, accelerate recovery and improve outcomes. This study aimed to investigate the feasibility and safety of combined exercise training and nutritional support in patients with haematologic malignancies prior to haematopoietic stem cell transplantation (HSCT).

**Methods:**

In this single-arm pilot study, 34 HSCT candidates were enrolled at least two weeks before admission for the procedure. Patients performed aerobic exercises at least 4 days per week for 20–30 min and strength exercises 3 days per week for 10–20 min. They received daily supplements of whey protein (0.3–0.4 g/kg body weight) and oral nutritional supplements if needed. The primary endpoints were feasibility (acceptability > 75%, attrition < 20%, adherence > 66%) and safety. The secondary endpoints were fat-free mass (FFM), muscle strength, physical performance and health-related quality of life (HRQoL) at HSCT.

**Results:**

The rate of acceptability, attrition and adherence to aerobic exercise, strength exercise and protein supplement consumption was 82.4, 17.8, 71, 78 and 80%, respectively. No severe adverse events were reported. Twenty-eight patients participated in the study for a median of 6.0 weeks (range, 2–14). They performed aerobic exercises 4.5 days per week for 132 min per week and strength exercises 3.0 times per week. Patients consumed 20.7 g of extra protein daily. At the end of the programme, we recorded increases of 1.1 kg in FFM (*p* = 0.011), 50 m in walking distance in the 6-min walking test (6MWT) (*p* < 0.001), 3.3 repetitions in the 30-s chair-stand test (30sCST) score (p < 0.001) and 2.6 kg in handgrip strength (*p* = 0.006). The EORTC QLQ-C30 scores improved by 8.6 (*p* < 0.006) for global health status, 8.3 (*p* = 0.009) for emotional functioning, and 12.1 (*p* = 0.014) for social functioning. There was less fatigue, nausea and insomnia (*p* < 0.05).

**Conclusions:**

Our study shows that a multimodal intervention programme with partially supervised exercise training combined with nutritional support prior to HSCT is feasible and safe. Patients showed improvements in FFM, physical performance and HRQoL. Additional research is needed to assess the possible positive effects of such interventions.

## Background

Haematopoietic stem cell transplantation (HSCT) is a standard of care for various adverse haematologic malignancies and improves the time to disease progression and patient overall survival [1–3]. The treatment outcome may be influenced by the underlying disease, age of the recipient, comorbidities, and allogeneic transplantation with a donor-recipient HLA mismatch [4]. The procedure can be accompanied by various complications [3]. The nutritional status [5, 6] and functional capacity [7, 8] of the patient have predictive value for the treatment outcome. In a study by Fuji et al., 14.8% of patients were underweight prior to HSCT, with a body mass index (BMI) < 18.5 kg/cm^2^, which was associated with worse overall survival [6]. Jones et al. evaluated the functional capacity in patients undergoing HSCT using the 6-min walking test (6MWT). Compared with that for patients achieving a distance of < 400 m, the unadjusted hazard ratio for overall survival was 0.71 for patients achieving a distance of ≥400 m. Each 50-m increase in distance was associated with a 9% relative reduction in the risk of death [8]. While the proportion of patients observed to have poor physical performance was as high as 46%, Wiskemann found that on average, patients showed a relatively good level of physical fitness on admission to the hospital but that the distance in the 6MWT decreased from an initial 546 m to 445 m on discharge [9].

Most of the associated factors cannot be addressed, but the implementation of certain measures may improve both the nutritional status and functional capacity of the patient. Prehabilitation was recently introduced to patients undergoing surgery for malignant disease, with the intention of improving functional capacity and nutritional status prior to the surgical procedure [10–13]. Preoperative exercise training and nutritional and psychological support improve the patient’s physiologic reserve prior to the surgical procedure. In patients with haematologic malignancies, exercise training in the post-HSCT period was shown to improve physical performance and muscle strength, reduce fatigue and improve health-related quality of life (HRQoL) [14, 15]. There are limited available data on the above measures prior to HSCT. In a pilot study, Bartels et al. reported on patients with lymphoma and multiple myeloma who safely performed a home-based exercise programme for an average of 11 weeks before HSCT [16]. Wiskemann et al. reported an improved survival rate at 2 years after allogeneic HSCT in a group of patients who enrolled in an exercise training programme for 21 days before, during, and 6 to 8 weeks following HSCT [17]. To prevent muscle protein depletion in cancer patients, combined nutrition and physical therapy are recommended [18]. While exercise stimulates muscle protein synthesis, protein intake can further improve training-induced muscle mass gains. Whey protein provides substantial amounts of essential amino acids and has proven to be effective in modulating postexercise muscle protein synthesis [13]. To the best of our knowledge, no data have been published on the effect of combined exercise training and nutritional support prior to HSCT. The goal of such an intervention would be for patients to increase their physiologic reserve in the anticipation of loss of muscle mass and impaired performance status during HSCT.

The purpose of our pilot study was to investigate the feasibility and safety of a multimodal intervention programme with partially supervised exercise training combined with nutritional support prior to HSCT.

## Methods

### Study design

In this single-arm pilot study, HSCT candidates were enrolled in an exercise training and nutritional support programme at least 2 weeks before planned admission to the University Medical Centre Ljubljana (UMCL) for HSCT. Admission was scheduled according to the type of HSCT, conditioning regimen and donor availability and was not influenced by participation in the study.

The study was approved by the Medical Ethics Committee of the Republic of Slovenia on 13.6.2017. All patients provided informed consent to participate in the study.

### Study objectives

The primary endpoints of the study were feasibility and safety. Based on previous studies [16] and the characteristics of our patient population, feasibility was considered to be achieved if the acceptability rate was > 75%, the attrition rate was < 20% and the adherence rate was > 66%. The secondary endpoints were fat-free mass (FFM), muscle strength, physical performance and health-related quality-of-life improvement at HSCT.

### Subjects

We included patients aged 18 years and above with haematologic malignancy who were scheduled for allogeneic or autologous HSCT. The exclusion criteria were hospitalization during the period immediately before HSCT, musculoskeletal disorders restricting physical activity, high risk of fracture due to osteolytic bone lesions, uncontrolled pain, unstable ischaemic heart disease, severe chronic obstructive pulmonary disease, exertional asthma and a known allergy to whey protein.

### Measurements

Measurements were performed at enrolment (T0) and on the day before commencing HSCT conditioning (T1). We performed a physical examination and blood tests as part of the standard procedure prior to HSCT. Patients were asked about their usual nutritional intake and proportion of protein in their diet. Each patient’s body mass (BM) and body height (BH) were measured, followed by BMI calculation. Body composition was measured using a bioimpedance method with a BIA 101^BM^ measuring device (Akern SRL, Montacchielo, Italy). We determined FFM, the fat-free mass index (FFMI), fat tissue mass (FM) and the fat tissue mass index (FMI). Indexed quantities (IQs) were calculated using the eq. IQ = measured quantity/BH^2^. Hand muscle strength (handgrip) was measured using a Jamar 5030^BM^ hand dynamometer (Sammons Preston, Inc., Bolingbrook, USA), and the average of the best left- and right-hand measurements was recorded. Physical performance was assessed with the 6MWT, in accordance with the American Thoracic Society guidelines [19], and the 30-s chair-stand test (30sCST), as previously described [20]. HRQoL was assessed using the validated European Organisation for Research and Treatment of Cancer Quality of Life Questionnaire-Core 30 (EORTC QLQ-C30) [21]. The HSCT-specific comorbidity index (HCT-CI) was calculated using the original tool developed by Sorror [22]. During the observational period, patients maintained an exercise training and nutrition diary. The physical activity of patients was also monitored with a Fitbit Charge 2^BM^ electronic sports bracelet (Fitbit, Inc., San Francisco, USA). The bracelet measures heart rate and counts steps using an accelerometer. We recorded the number of daily steps and the duration of physical activity above 3 metabolic equivalents of task (METs). The heart rate reserve (HRR) was calculated using the equation HRR (min^− 1^) = 220 - age (years) - pulse at rest (min^− 1^).

### Exercise training programme

A physician and a physiotherapist designed an exercise training programme for each patient individually, considering the initial fitness level and the progress made during the study period. The exercise was in line with the recommendations for mild to moderate intensity [23, 24]. Patients were instructed to exercise at home and weekly at the UMCL facility under the supervision of a physiotherapist. During the weekly supervised exercise, the physician and the physiotherapist checked the exercise technique and adjusted the exercise plan.

Patients were instructed to perform aerobic exercises by exercising on a bicycle, walking fast or walking with poles (i.e., Nordic walking) at least 4 days per week for 20–30 min, for a total of 100 min per week minimum. The target heart rate during the activity was 40–70% HRR. Patients used electronic sport bracelets to monitor their heart rate and therefore control the exercise intensity. The intensity and duration of the exercise increased at a weekly interval according to the following scheme: in the first week, exercise lasted 20 min with a heart rate of 40–60% HRR; in the second week, exercise lasted 30 min with a heart rate of 40–60% HRR; in the third week, exercise lasted 20 min with a heart rate of 50–70% HRR; and from the fourth week onwards, exercise lasted 30 min with a heart rate of 50–70% HRR.

Patients were instructed to perform strength exercises 3 days per week for 10–20 min daily. The training consisted of 1–3 series of 6–15 repetitions of the chair-stand exercise, the wall leaning exercise and exercises with elastic bands for the flexors and extensors of the elbows, knees and hips. The number of repetitions of the exercises and the resistance of the elastic bands increased at a weekly interval.

Before each training session, patients warmed up briefly, and after the training session, cool down and stretching exercises were performed (5–10 min).

In cases of uncertainty, patients could consult the responsible investigator and discontinue the exercise if indicated. Patients discontinued the training programme in the event of an infection, severe anaemia (Hb < 80 g/L), neutropenia (ANC < 0.5 × 10^6^/L), thrombocytopenia (Tr < 30 × 10^6^/L), coagulation disorder (INR > 1.5 or PTT > 50 s), and metabolic or electrolytic disorder (i.e., glucose > 12 mmol/L, Na < 135 mmol/L or Na > 145 mmol/L, K < 3.8 mmol/L or K > 5.5 mmol/L, or a creatinine increase by more than 50 μmol/L above the baseline value).

### Nutritional support

If there were no personal dietary restrictions, each patient’s baseline daily protein intake was estimated to be 1.0 g/kg. During the study period, patients received a daily supplement of 0.3–0.4 g/kg body weight of whey protein (Fresubin Protein Powder®, Fresenius Kabi, Bad Homburg, Germany) to reach the recommended protein intake of 1.0–1.5 g/kg/day [18]. Patients were instructed to consume protein supplements shortly after exercise.

Nutritionally deprived patients (i.e., those with BMI < 20, weight loss or a food intake problem) were also offered an oral nutritional supplement containing 300 kcal and 20 g of protein (Fresubin protein energy®, Fresenius Kabi, Bad Homburg, Germany).

### Statistical analysis

To assess feasibility, we calculated the proportion of eligible patients who agreed to participate in the study (acceptability). We then calculated the proportion of patients who stopped participating in the study before its completion (attrition). Adherence to the study schedule was evaluated from each patient’s diary and electronic bracelet data. We calculated the proportion of patients who completed at least the minimum prescribed amount of exercise (100 min of aerobic exercises and 2.5 strength exercises per week) and the proportion of patients who consumed at least 67% of the prescribed amount of protein powder. We recorded all the complications observed during the study. To assess the changes in the secondary endpoints, the average values and standard deviation (SD) of the measured quantities were calculated at the beginning (T0) and at the end (T1) of the study period. Differences were determined by paired t-test, and a *P* value < 0.05 was considered significant.

## Results

### Feasibility and safety

**Fig. 1:**
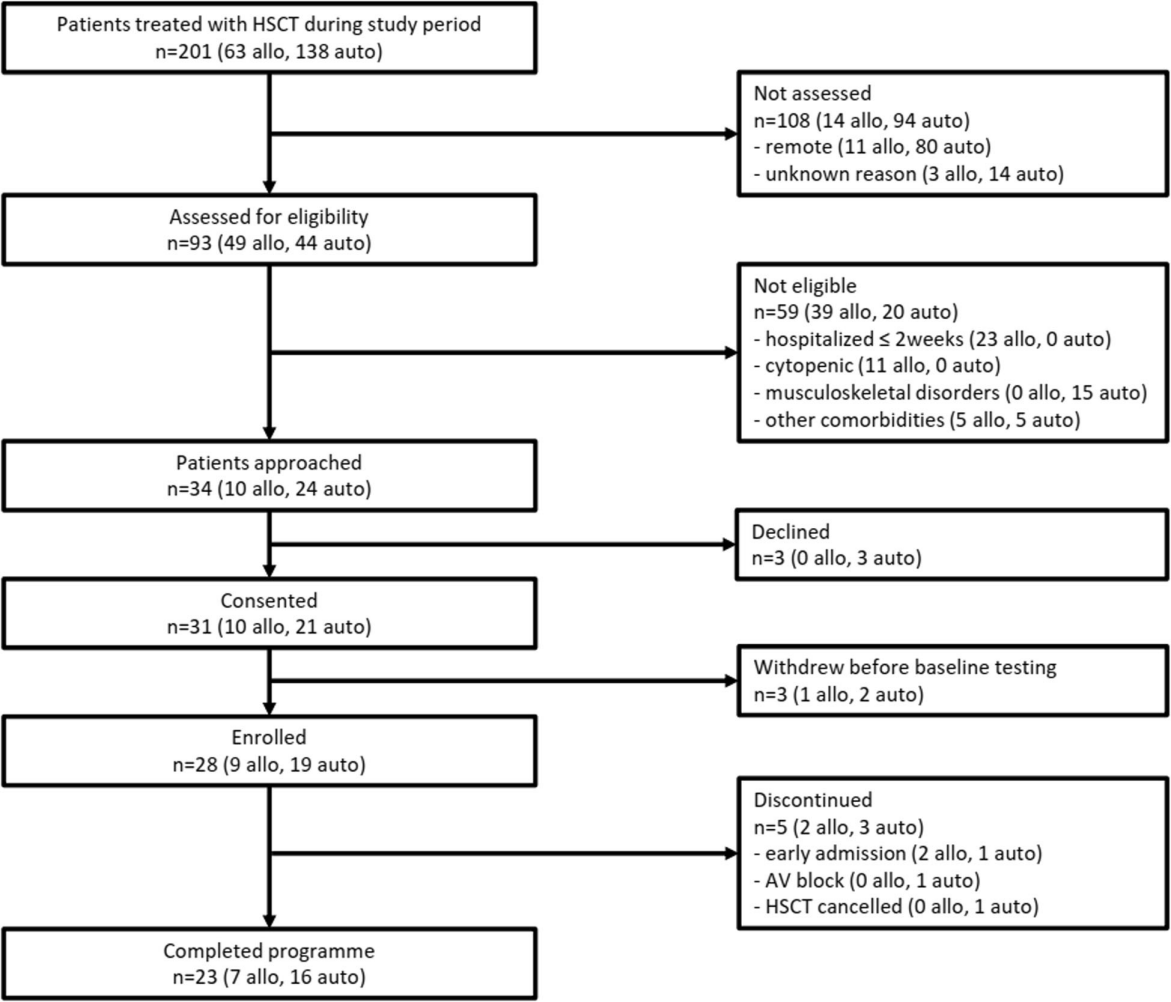
Patient flow diagram

From January 2018 to July 2019, 34 patients were enrolled, and 28 patients agreed to participate (acceptability, 82.4%). Patient flow during the study is presented in Fig. 1. The average patient age was 59.4 (±8.2) years; 10 were female, and 18 were male; 9 (32%) were scheduled for allogeneic HSCT, and 19 (68%) were scheduled for autologous HSCT (Table 1).

**Table 1:** Patient characteristics included in the study and of the patients who declined to participate

	Participants (***n*** = 28)	Non-participants (***n*** = 6)
**Age, years**		
Mean (SD)	59.4 (8.2)	60.7 (12.7)
**Sex (n, %)**		
Male	18 (64)	4 (67)
Female	10 (36)	2 (33)
**BMI, kg/m**^**2**^		
Mean (SD)	25.0 (3.6)	25.6 (2.7)
**BMI, kg/m**^**2**^ **(n, %)**		
BMI < 20	1 (4)	0 (0)
BMI 20–25	15 (53)	2 (33)
BMI 25–30	7 (25)	4 (67)
BMI > 30	5 (18)	0 (0)
**HCT-CI (n, %)**		
0	13 (46)	4 (67)
1–2	9 (32)	2 (33)
≥ 3	6 (21)	0 (0)
**ECOG performance status (n, %)**		
0	6 (21)	1 (17)
1	13 (46)	4 (67)
2	9 (32)	1 (17)
**Diagnosis (n, %)**		
MM	16 (57)	5 (83)
AML	6 (21)	0 (0)
ALL	2 (7)	0 (0)
Amyloidosis	2 (7)	0 (0)
MCL	1 (4)	0 (0)
MDS	1 (4)	0 (0)
Primary myelofibrosis	0 (0)	1 (17)
**Treatment history**		
Bortezomib- or IMID-based induction	18 (64)	5 (83)
Intensive chemotherapy (DA or HD ARA-C)	7 (25)	0 (0)
Rituximab-Bendamustine	1 (4)	0 (0)
Azacitidine	1 (4)	0 (0)
Methotrexate (ALL maintenance)	1 (4)	0 (0)
Ruxolitinib	0 (0)	1 (17)
**HSCT type (n, %)**		
Autologous	19 (68)	5 (83)
Allogeneic	9 (32)	1 (17)

*BMI* body mass index; *HCT-CI* haematopoietic cell transplantation-specific comorbidity index; *MM* multiple myeloma; *AML* acute myeloid leukaemia; *ALL* acute lymphoblastic leukaemia; *MCL* mantle cell lymphoma; *MDS* myelodysplastic syndrome; *IMID* immunomodulatory drug; *DA* daunorubicin and cytarabine; *HD ARA-C* high-dose cytarabine; *HSCT* haematopoietic stem cell transplantation

Five patients completed the programme early (attrition, 17.8%). Three patients discontinued the study due to expedited admission for HSCT, one patient discontinued because HSCT was cancelled due to an insufficient collection of stem cells, and one patient discontinued due to a grade III AV heart block. The block occurred while resting and was not considered an adverse event linked to the prehabilitation programme. Following pacemaker implantation, the patient underwent autologous HSCT. No other complications were observed during the study.

There were no serious adverse events due to exercise or protein intake. Two patients reported mild transient muscle soreness, one patient showed fluoroquinolone-associated tendinopathy, and one patient showed lower leg cellulitis. Four patients reported worsening of multiple myeloma-associated skeletal pain, and two showed worsening of a chronic arthritic condition. Two patients showed worsening of peripheral neuropathy. Two patients transiently omitted exercises due to upper respiratory tract viral infections, and one patient did so due to viral diarrhoea. Protein supplements were generally well tolerated. Patients with a history of gastric surgery showed early satiety, and another three patients reduced their prescribed quantity of protein consumption due to bloating or gastric discomfort.

Patients performed the programme for an average of 6.8 weeks (median, 6.0; range, 2–14). The rate of participation in guided exercises averaged 73%, with at least two-thirds participation among 68% of patients. According to the diary data, patients performed aerobic exercises for an average of 4.5 days per week and for an average duration of 132 min per week. A total of 67% of patients adhered to the aerobic exercise schedule at least 4 days per week, and 71% adhered to the aerobic exercise duration of at least 100 min per week. Patients performed the strength exercises on average 3.0 times per week. Seventy-eight percent of patients performed strength exercises at least 2.5 times per week. According to the sports bracelet data, patients took an average of 7416 steps per day and had at least 45 min of moderately intense physical activity (≥3 METs) per day (Table 2).

**Table 2:** Physical activity and nutrition

Duration of programme, weeks	6.8 (3.9)
**Daily activity**	
Steps per day	7416 (2893)
Activity ≥3 METs, minutes per day	45 (20)
**Supervised exercise**	
Sessions planned	6.8 (2.1)
Sessions attended	5.0 (3.3)
**Unsupervised exercise**	
Weekly performed aerobic exercises, days	4.5 (1.5)
Weekly performed aerobic exercises, min	132 (67)
Weekly performed strength training, days	3.0 (0.7)
**Oral nutritional supplements**	
Protein powder prescribed, g/day (mean)	24.2 (5.3)
Protein powder prescribed, g/kg/day (mean)	0.32 (0.03)
Protein powder consumed, g/kg/day (mean)	0.28 (0.08)
Caloric supplement prescribed, patients	2
Caloric supplement consumed, patients	0

Values are presented as the mean (SD). METs, metabolic equivalents of task

Most HSCT candidates were not at nutritional risk. Poor food intake and weight loss were reported by two patients, one with amyloidosis and one after gastric surgery. Both were offered an oral nutritional supplement that was poorly tolerated. After diet modification, their energy intake was sufficiently increased. The participants were prescribed an average of 24.2 g of a protein powder supplement daily, which equalled 0.32 g/kg of BM daily. On average, they consumed 20.7 g of extra protein daily, which is 85% of the intended amount. Eighty percent of participants completed the nutritional support plan and consumed at least 67% of the prescribed amount of protein powder (Table 2).

### Secondary endpoints

At the end of the programme, we found an average increase in FFM of 1.1 kg (*p* = 0.011) (Table 3). The average walking distance in the 6MWT increased from 520 m to 570 m (*p* < 0.001). The average score on the 30sCST test increased from 12.7 repetitions to 16.0 repetitions (p < 0.001). The average handgrip strength increased from 34.9 kg to 37.5 kg (*p* = 0.006) (Table 4). The EORTC QLQ-C30 showed an improvement in the global health status score from an average of 56.5 to 65.1 (*p* < 0.006), an improvement in the emotional functioning score from 77.3 to 85.6 (*p* = 0.009), and an improvement in the social functioning score from 61.4 to 73.5 (*p* = 0.014). Additionally, there was less fatigue (40.0 to 26.6, *p* = 0.004), nausea (6.9 to 3.8, *p* = 0.042) and insomnia (36.4 to 25.6, *p* = 0.015) after the programme (Table 5).

**Table 3:** Changes in BMI and body composition

	T0	T1	p
BMI, kg/m^2^	25.0 (3.6)	25.3 (3.6)	0.014
FFM, kg	56.7 (10.2)	57.8 (9.5)	0.011
FM, kg	16.9 (5.6)	17.0 (5.6)	0.836

Values are presented as the mean (SD). *BMI* body mass index; *FFM* fat-free mass; *FM* fat mass

**Table 4:** Changes in physical performance and strength

	T0	T1	p
6MWT, m	520 (89)	570 (91)	< 0.001
30sCST	12.7 (3.5)	16.0 (4.7)	< 0.001
Handgrip, kg	34.9 (9.6)	37.5 (9.3)	0.006

Values are presented as the mean (SD). 6MWT, 6-min walking test; 30sCST, 30-s chair-stand test

**Table 5:** Changes in HRQoL (EORTC QLQ-C30)

	T0	T1	p
**GLOBAL HEALTH STATUS**	56.5 (20.5)	65.1 (22.4)	0.006
**FUNCTIONAL SCALES**			
Physical functioning	76.6 (17.5)	81.8 (22.9)	0.115
Role functioning	62.8 (35.6)	73.5 (28.9)	0.093
Emotional functioning	77.3 (21.2)	85.6 (18.8)	0.009
Cognitive functioning	80.4 (20.9)	87.1 (22.9)	0.072
Social functioning	61.4 (31.4)	73.5 (28.0)	0.014
**SYMPTOM SCALES**			
Fatigue	40.0 (24.1)	26.6 (28.2)	0.004
Nausea/vomiting	6.9 (11.1)	3.8 (10.1)	0.042
Pain	28.0 (28.3)	22.0 (24.3)	0.180
Dyspnoea	12.0 (19.3)	13.5 (16.6)	0.591
Insomnia	36.4 (34.1)	25.6 (30.7)	0.015
Appetite loss	13.6 (24.5)	12.1 (26.3)	0.660
Constipation	19.7 (33.6)	10.6 (29.8)	0.083
Diarrhoea	1.5 (7.0)	3.0 (9.7)	0.576
Financial difficulties	16.6 (28.7)	22.7 (34.8)	0.103

Values are presented as the mean (SD)

## Discussion

Our study shows that exercise training and nutritional support protocols were feasible and safe in patients prior to HSCT. Of the eligible patients in the study, most (82.4%) accepted enrolment. Most participants successfully completed the programme, with only 17.8% leaving the programme early. Based on participation in the guided exercises, the diary data and the data recorded by sports bracelets, adherence to the protocol was above expectations. Although more than half of the patients had accompanying diseases and one-third of the patients had a poor performance status, most patients fulfilled the planned range of exercises. According to data from the diaries, almost all patients performed strength exercises regularly, three times per week. They were slightly less engaged in aerobic exercises but still performed them satisfactorily on average. Weekly controlled training at the facility, with added professional supervision and adjustment of the exercise routine, proved to be pivotal for good adherence in our patient group. The supervision of exercise techniques is also important to decrease the risk of injury and possible adverse events.

Due to chronic systemic inflammation and metabolic changes, patients with malignancies already experience an increased need for protein intake [18]. The data on the occurrence of malnutrition and underweight in haematologic patients show an association with reduced overall survival and increased rates of relapse and transplant-related mortality in some studies, while others do not show any correlation [25]. In the paediatric population, optimizing the nutritional status prior to HSCT is of great importance [26]. At least one study reported a prevalence of sarcopenia in the adult population of up to 50.6% prior to allogeneic HSCT [27]. Since an increase in muscle mass was our goal, we prescribed a protein supplement. The addition of physical exercise to protein supplementation was not performed in haematologic patients prior to HSCT. However, in surgery patients, the combination of exercise and protein supplementation is synergistic. The relative increase in muscle strength after bariatric surgery was higher in patients performing strength exercises in combination with protein supplementation than in patients only receiving protein supplementation [28]. Protein supplementation improved muscle strength and physical function more than exercise alone in ageing patients with sarcopenia [29]. There is accumulating evidence that the combination of strength exercises with nutritional support is beneficial in patients undergoing surgery or in critically ill patients with a risk of sarcopenia. In a systematic review, multimodal prehabilitation was safe with good adherence even in high-risk patients awaiting lung or heart transplantation [30]. Protein powder was well tolerated, and at the end of the study, we measured an increase in the FFM of the participants. The patients’ physical fitness at the beginning of the programme was good. The number of patients who were able to walk less than 400 m in the 6MWT, which is a risk factor for a poor HSCT outcome, was only 3 [8]. In one patient, the reason for the poor 6MWT result was peripheral neuropathy due to the underlying disease, and the other two patients performed poorly because they had just completed their last round of chemotherapy. On average, we measured better participant outcomes at the end of the programme, namely, for the 6MWT, as well as for the 30sCST and handgrip test. According to the EORTC QLQ-C30 results, patients experienced improvements in quality of life at the end of the programme. In general, they felt less ill and more emotionally balanced, and they reported less fatigue, less nausea and less insomnia. Whether this was the result of our prehabilitation programme or other factors remains to be assessed in future controlled studies.

There were no serious adverse events due to exercise or protein intake. If patients omitted aerobic exercise for a short period, they mostly reported only general malaise. Other reasons for omitting exercises were upper respiratory tract viral infections or poor weather. None of the participants terminated the programme due to limitations in blood counts or electrolyte or metabolic disturbance. No patient with multiple myeloma with bone involvement developed any musculoskeletal injuries.

Our study had some limitations. First, it was not a controlled randomized study, so we were not able to demonstrate that the positive effects were due to our intervention. Since we showed that the intervention is feasible and safe, we plan to continue and expand the study in the future. Second, patient enrolment in the study was slow, which resulted in a small patient sample. Because screening was limited to patients who could perform the programme for at least 2 weeks at home and attend the guided group exercises once per week, we were not able to screen most patients planning to undergo allogeneic HSCT, since the time to HSCT was on average very short. Recently hospitalized patients in particular could benefit from the intervention and will be included in future studies as soon as they become candidates for HSCT. Patients from remote locations were also not screened for the study. Regarding promising results from our feasibility study, we intend to increase our resources to be able to include these patients in the future. Third, body composition was measured with bioimpedance, which is an indirect method. Bioimpedance is less accurate than dual-energy X-ray absorptiometry, computed tomography or magnetic resonance imaging. However, bioimpedance is readily available, safe and commonly used in everyday clinical practice [31].

## Conclusions

Our study shows that a multimodal intervention programme with partially supervised exercise training combined with nutritional support prior to HSCT is feasible and safe. Patients who participated in the programme showed improved body composition through increases in FFM, improved physical performance, and improved HRQoL. Further randomized trials are needed to examine the impact of multimodal interventions on quality of life, body composition, disease progression and, potentially, overall patient survival.

## Data Availability

The datasets used and/or analysed during the current study are available from the corresponding author on reasonable request.

## Abbreviations

HSCT: Haematopoietic stem cell transplantation
HRQoL: Health-related quality of life
BM: Body mass
BMI: Body mass index
BH: Height
FFM: Fat-free mass
FFMI: Fat-free mass index
FM: Fat mass
FMI: Fat mass index
6MWT: 6-min walking test
30sCST: 30-s chair-stand test
HCT-CI: Haematopoietic cell transplantation-specific comorbidity index
EORTC QLQ-C30: European Organisation for Research and Treatment of Cancer Quality of Life Questionnaire-Core 30
METs: Metabolic equivalents of task
ECOG: Eastern Cooperative Oncology Group
HRR: Heart rate reserve
Hb: Haemoglobin
ANC: Absolute neutrophil count
Tr: Thrombocytes
INR: International normalized ratio
PTT: Partial thromboplastin time
MM: Multiple myeloma
AML: Acute myeloid leukaemia
ALL: Acute lymphoblastic leukaemia
MCL: Mantle cell lymphoma
MDS: Myelodysplastic syndrome
IMID: Immunomodulatory drug
DA: Daunorubicin and cytarabine
HD ARA-C: High-dose cytarabine
UMCL: University Medical Centre Ljubljana
